# The high-risk features and effect of postoperative radiotherapy on survival for patients with surgically treated stage IIIA-N2 non-small cell lung cancer

**DOI:** 10.1186/s12957-023-03093-8

**Published:** 2023-08-04

**Authors:** Minxia Zhu, Shaomin Li, Liyue Yuan, Shiyuan Liu, Jianzhong Li, Danjie Zhang, Jia Chen, Jiantao Jiang, Zhengshui Xu

**Affiliations:** 1 Department of Thoracic Surgery, The Second Affiliated Hospital of Xi’an Jiaotong University, Xi’an, 710004 Shaanxi China; 2 Department of Thoracic Surgery, The First Affiliated Hospital of Wenzhou Medical University, Wenzhou, 325000 Zhejiang China; 3 Department of Endocrinology, The First Affiliated Hospital of Xi’an Jiaotong University, Xi’an, 710061 Shaanxi China; 4 Department of Cardiovascular Surgery, Xijing Hospital, Fourth Military Medical University, Xi’an, 710032 Shaanxi China

**Keywords:** Non-small cell lung cancer, Post-operative radiotherapy, Mediastinal involvement, Overall survival, Cancer special survival

## Abstract

**Objectives:**

Although postoperative radiotherapy (PORT) could reduce the incidence of local recurrence in patients with IIIA-N2 non-small cell lung cancer (NSCLC), the role of PORT on survival in patients with surgically treated stage IIIA-N2 NSCLC remains controversial. Therefore, this study was designed to evaluate the effect of PORT on survival for patients with surgically treated stage IIIA-N2 NSCLC.

**Materials and methods:**

This study population was chosen from the Surveillance, Epidemiology, and End Results database. The Cox proportional hazards regression analysis was used to determine significant contributors to overall survival (OS) and cancer special survival (CSS) outcomes. To balance baseline characteristics between the non-PORT group and PORT group, propensity score matching (PSM) with 1:1 propensity nearest-neighbor match by 0.001 matching tolerance was conducted by R software. Furthermore, a Kaplan–Meier curve was used to visualize the OS and CSS between the PORT group and non-PORT group survival probability.

**Results:**

Of all evaluated cases, 4511 with IIIA-N2 NSCLC were eligible for inclusion, of which 1920 were enrolled into the PORT group. On univariate analysis and multivariate analysis, sex, age, year of diagnosis, race, histologic type, T stage, PORT, use of chemotherapy, and positive regional nodes were significantly associated with OS and CSS in IIIA-N2 NSCLC (*P* < 0.05). However, PORT was not significantly associated with OS (univariate HR = 0.92, 95%CI 0.85–0.99, *P* = 0.02; multivariate HR = 1.01, 95%CI 0.93–1.08, *P* = 0.91) and CSS (univariate HR = 0.92, 95%CI 0.85–1.01, *P* = 0.06; multivariate HR = 1.103 95%CI 0.94–1.12, *P* = 0.56) in IIIA-N2 NSCLC. Meanwhile, after PSM, neither OS nor CSS did differ significantly between the non-PORT group and PORT group (OS HR = 1.08, 95%CI 0.98–1.19, *P* = 0.12; CSS HR = 1.10, 95%CI 0.99–1.23, *P* = 0.07).

**Conclusion:**

PORT did not contribute to a survival benefit in patients with surgically treated stage IIIA-N2 NSCLC.

**Supplementary Information:**

The online version contains supplementary material available at 10.1186/s12957-023-03093-8.

## Introduction

Lung cancer is the second most commonly diagnosed cancer and the leading cause of cancer-related lethality globally [1]. Non-small cell lung cancer (NSCLC) accounts for about 85% of patients with lung cancer, and one-third have been diagnosed with locally advanced NSCLC [2]. Surgery-based multimodality therapies are one of the available curative treatments for operable advanced NSCLC, especially pathological N2 IIIA NSCLC [3–5]. Notably, the role of PORT in the survival of patients with stage IIIA-N2 NSCLC has caused considerable controversy [3, 4]. It is widely revealed that PORT contributes to a significant benefit in local–regional control in patients with surgically treated stage IIIA-N2 NSCLC [3, 4, 6, 7]. However, the benefit of local–regional control did not stand for an OS advantage. Additionally, PORT naturally increases adverse events, such as cardiopulmonary toxicity [3, 8, 9]. In general, the role of PORT in patients with surgically treated stage IIIA-N2 NSCLC remains controversial [3, 4]. Therefore we aimed to assess the role of PORT on the survival of patients with stage IIIA-N2 NSCLC by a Surveillance, Epidemiology, and End Results Program (SEER) population-based study.

## Methods

Data were extracted from the SEER database, a national registry funded by the National Cancer Institute since 1971. This study population was chosen from the SEER Research Plus Database (17 Regs, Nov 2021Sub [2000–2019]) using SEER*stat Version 8.4.0.1 software. Patients who were 18 years old or more diagnosed with primary IIIA-N2 NSCLC were included according to the International Classification of Diseases for Oncology, 3rd edition morphological code including adenocarcinoma (SEER code 8140–8143, 8211, 8230 8250–8255 8323 8480 8481 8490 8550 8570,8572,8574), squamous cell carcinoma (SEER code 8050, 8052, 8070–8078, 8083, 8084, 8123), and others (SEER code 8012–8014, 8046, 8003, 8004, 8022, 8030–8032, 8200, 8240, 8249, 8560). Furthermore, we checked and reclassified the stage IIIA-N2 NSCLC according to the latest 8th Edition of American Joint Committee on Cancer (AJCC) TNM staging system instead of the TNM staging system by SEER [10].

### Data extraction, collection, and exclusion

**Fig. 1:**
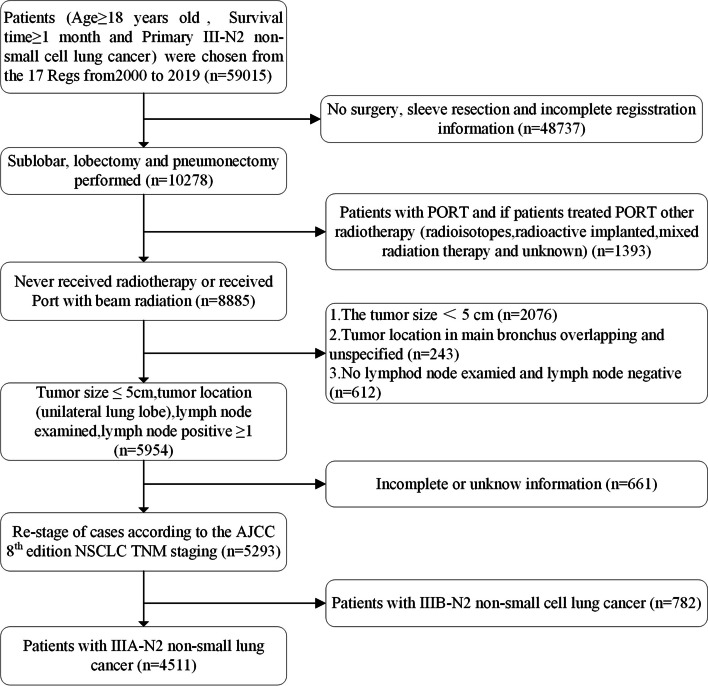
Patient selection for this study

The eligibility criteria of this study and the workflow are presented in Fig. 1. Patients with surgically treated (who perform lobectomy or pneumonectomy) stage IIIA-N2 NSCLC were classified as whether they had a PORT (according to code, received beam radiation after surgery or without radiotherapy). The relevant and complete information was extracted as follows, sex, age, year of diagnosis, race, primary site, laterality, histologic type, tumor size, T stage, surgical procedure, use of radiotherapy, use of chemotherapy, positive regional nodes, presumed survival in months, vital status, and cause of death.

### Statistical analysis

Categorical variables are presented as numbers and percentages. Chi-square tests and *t* tests were performed for descriptive analyses. The Cox proportional hazards model was used to determine significant contributors from potential prognostic factors, including age (divided into 2 groups of 65 years old), sex, race, year of diagnosis (divided into 2 periods of 10 years each [2000–2009 2010–2019]), primary site, laterality, histologic type, T stage, use of PORT, use of chemotherapy and positive regional nodes to CSS and OS outcomes. To balance baseline characteristics between non-PORT and PORT groups, R software conducted propensity score matching (PSM) with 1:1 propensity nearest-neighbor match by 0.001 matching tolerance. A Kaplan–Meier curve was used to visualize the OS and CSS between PORT and Non-PORT survival probability. The result visualization by performing R software (Version 3.5.1, package “survival”, “MatchIt”). All P values were 2-sided, with a *p* value < 0.05 were considered statistically significant.

## Results

### Patient characteristics

In total, 4511 cases with stage IIIA-N2 NSCLC were eligible for inclusion between 2000 and 2019 (Fig. 1), of which 1920 cases were enrolled into the PORT group (Table 1). The number and proportion of surgically treated stage IIIA-N2 NSCLC following PORT significantly increased over the last two decades. The rate of older (age ≥ 65) patients who received surgically treated stage IIIA-N2 NSCLC following PORT decreased obviously during the last two decades of age.

**Table 1:** Characteristics of patients with stage IIIA-N2 NSCLC before PSM

Characteristic	Non-PORT	PORT	*P* value
n	2591	1920	
Age, *n* (%)			< 0.01
< 65	994 (22%)	938 (20.8%)	
≥ 65	1597 (35.4%)	982 (21.8%)	
Sex, *n* (%)			0.87
Female	1400 (31%)	1032 (22.9%)	
Male	1191 (26.4%)	888 (19.7%)	
Year of diagnosis, *n* (%)			0.02
2000–2009	1288 (28.6%)	884 (19.6%)	
2010–2019	1303 (28.9%)	1036 (23%)	
Race, *n* (%)			0.70
White	2110 (46.8%)	1554 (34.4%)	
Other	481 (10.7%)	366 (8.1%)	
Primary site, *n* (%)			0.01
Upper lobe	1579 (35%)	1245 (27.6%)	
Middle lobe	125 (2.8%)	99 (2.2%)	
Lower lobe	887 (19.7%)	576 (12.8%)	
Laterality, *n* (%)			0.98
Left	1198 (26.6%)	886 (19.6%)	
Right	1393 (30.9%)	1034 (22.9%)	
Histologic type, *n* (%)			< 0.01
LUAD	1742 (38.6%)	1411 (31.3%)	
LUSC	511 (11.3%)	308 (6.8%)	
Large cell	85 (1.9%)	61 (1.4%)	
Other	253 (5.6%)	140 (3.1%)	
T stage, *n* (%)			0.03
T1	989 (21.9%)	796 (17.6%)	
T2	1602 (35.5%)	1124 (24.9%)	
Surgery, *n* (%)			< 0.01
Lobectomy	2187 (48.5%)	1586 (35.2%)	
Sublobectomy	229 (5.1%)	242 (5.4%)	
Pneumonectomy	175 (3.9%)	92 (2%)	
Chemotherapy, *n* (%)			< 0.01
No	1173 (26%)	282 (6.3%)	
Yes	1418 (31.4%)	1638 (36.3%)	
Regional nodes positive, *n* (%)			< 0.01
4	1745 (38.7%)	1192 (26.4%)	
≥ 4	846 (18.8%)	728 (16.1%)	

### Survival and high-risk features

On univariate analysis and multivariate analysis, age, year of diagnosis, sex, race, histologic type, T stage, use of chemotherapy, and positive regional nodes were significantly associated with OS and CSS in stage IIIA-N2 NSCLC (*P* < 0.05, Tables 2 and 3). From multivariate analysis, PORT was not significantly associated with OS (HR = 1.01, 95%CI 0.93–1.08, *P* = 0.91) and CSS (HR = 1.103 95%CI 0.94–1.12, *P* = 0.56) in stage IIIA-N2 NSCLC (Tables 2 and 3).

**Table 2:** Univariate and multivariate analysis of OS for patients with stage IIIA–N2 NSCLC

Characteristics	Total (*N*)	Univariate analysis		Multivariate analysis	
		Hazard ratio (95% CI)	*P* value	Hazard ratio (95% CI)	*P* value
Age	4511				
< 65	1932	Reference			
≥ 65	2579	1.43 (1.33–1.54)	< 0.01	1.35 (1.25–1.45)	< 0.01
Sex	4511				
Female	2432	Reference			
Male	2079	1.34 (1.25–1.44)	< 0.01	1.32 (1.23–1.42)	< 0.01
Year of diagnosis	4511				
2000–2009	2172	Reference			
2010–2019	2339	0.67(0.61–0.71)	< 0.01	0.69 (0.64–0.75)	< 0.01
Race	4511				
White	3664	Reference			
Other	847	0.80 (0.73–0.88)	< 0.01	0.82 (0.75–0.91)	< 0.01
Primary site	4511				
Upper lobe	2824	Reference			
Middle lobe	224	1.04 (0.88–1.22)	0.68	1.18 (0.99–1.40)	0.06
Lower lobe	1463	1.06 (0.98–1.14)	0.17	1.07 (0.99–1.16)	0.08
Laterality	4511				
Left	2084	Reference			
Right	2427	0.95 (0.89–1.02)	0.15	0.97 (0.91–1.05)	0.49
Histologic type	4511				
LUAD	3153	Reference			
LUSC	819	1.24 (1.13–1.36)	< 0.01	1.10 (1.00–1.21)	0.05
Large cell	146	1.25 (1.03–1.51)	0.02	1.19 (0.99–1.44)	0.07
Other	393	1.01 (0.88–1.15)	0.92	0.98 (0.86–1.12)	0.78
T stage	4511				
T1	1785	Reference			
T2	2726	1.24 (1.15–1.33)	< 0.01	1.23 (1.14–1.32)	< 0.01
Surgery	4511				
Lobectomy	3773	Reference			
Sublobectomy	471	1.29 (1.15–1.44)	< 0.01	1.38 (1.23–1.54)	< 0.01
Pneumonectomy	267	1.27 (1.10–1.46)	< 0.01	0.99 (0.85–1.15)	0.89
Surg/Rad Seq	4511				
Non-PORT	2591	Reference			
PORT	1920	0.92 (0.85–0.99)	0.02	1.01 (0.93–1.08)	0.91
Chemotherapy	4511				
No	1455	Reference			
Yes	3056	0.66 (0.61–0.71)	< 0.01	0.72 (0.66–0.78)	< 0.01
Regional nodes positive	4511				
< 4	2937	Reference			
≥ 4	1574	1.36 (1.27–1.47)	< 0.01	1.48 (1.37–1.59)	< 0.01

**Table 3:** Univariate and multivariate analysis of CSS for patients with stage IIIA–N2 NSCLC

Characteristics	Total (*N*)	Univariate analysis		Multivariate analysis	
		Hazard ratio (95% CI)	*P* value	Hazard ratio (95% CI)	*P* value
Age	3881				
< 65	1708	Reference			
≥ 65	2173	1.46 (1.35–1.59)	< 0.01	1.37 (1.26–1.49)	< 0.01
Sex	3881				
Female	2132	Reference			
Male	1749	1.39 (1.28–1.50)	< 0.01	1.37 (1.26–1.49)	< 0.01
Year of diagnosis	3881				
2000–2009	1747	Reference			
2010–2019	2134	0.54 (0.50–0.59)	< 0.01	0.56 (0.52–0.61)	< 0.01
Race	3881				
White	3126	Reference			
Other	755	0.78 (0.70–0.86)	< 0.01	0.81 (0.73–0.90)	< 0.01
Primary site	3881				
Upper lobe	2421	Reference			
Middle lobe	202	1.01 (0.84–1.21)	0.92	1.17 (0.97–1.41)	0.10
Lower lobe	1258	1.04 (0.95–1.13)	0.44	1.06 (0.97–1.160)	0.18
Laterality	3881				
Left	1764	Reference			
Right	2117	0.94 (0.87–1.02)	0.11	0.94 (0.88–1.02)	0.14
Histologic type	3881				
LUAD	2777	Reference			
LUSC	658	1.30 (1.17–1.45)	< 0.01	1.17 (1.04–1.30)	0.01
Large cell	114	1.34 (1.07–1.67)	0.01	1.25 (1.00–1.57)	0.05
Other	332	1.01 (0.87–1.17)	0.91	1.00 (0.86–1.16)	0.99
T stage	3881				
T1	1532	Reference			
T2	2349	1.26 (1.16–1.37)	< 0.01	1.260 (1.16–1.37)	< 0.01
Surgery	3881				
Lobectomy	3259	Reference			
Sublobectomy	405	1.33 (1.17–1.50)	< 0.01	1.45 (1.27–1.64)	< 0.01
Pneumonectomy	217	1.31 (1.12–1.53)	< 0.01	0.95 (0.80–1.13)	0.55
Surg/Rad Seq	3881				
Non-PORT	2214	Reference			
PORT	1667	0.92 (0.85–1.01)	0.06	1.03 (0.94–1.12)	0.56
Chemotherapy	3881				
No	1180	Reference			
Yes	2701	0.61 (0.56–0.66)	< 0.01	0.70 (0.64–0.77)	< 0.01
Regional nodes positive	3881				
< 4	2505	Reference			
≥ 4	1376	1.40 (1.29–1.51)	< 0.01	1.51 (1.38–1.64)	< 0.01

**Fig. 2:**
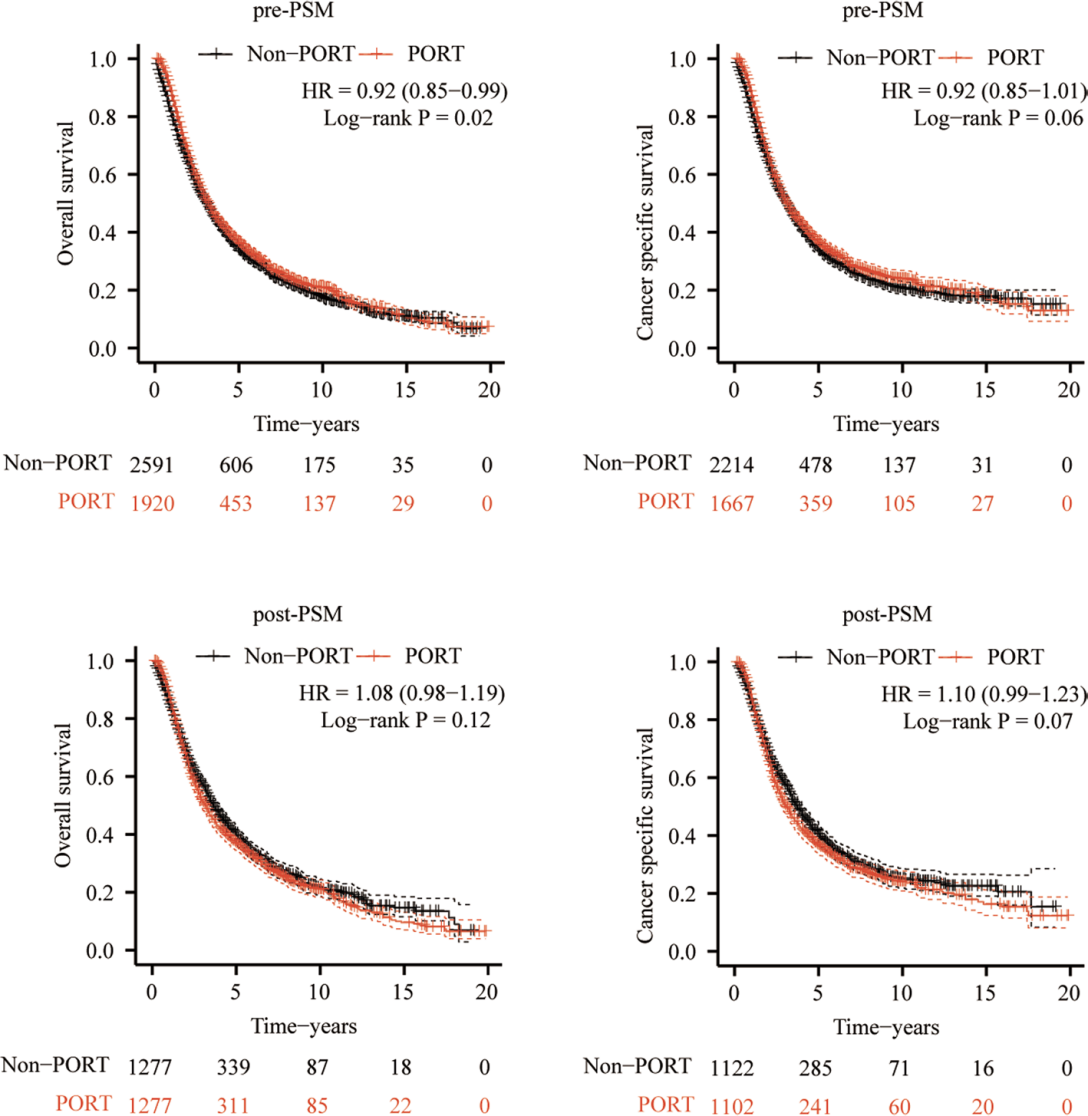
Kaplan–Meier curves of the OS and CSS between PORT and non-PORT before and after PSM. **A** OS curves before PSM. **B** CSS curves before PSM. **C** OS curves after PSM. **D** CSS curves after PSM. OS: overall survival, CSS: cancer-specific survival, PSM: propensity score-matching

Prior to PSM, PORT might contribute to an OS benefit (HR = 0.92, 95% CI 0.85–0.99, *P* = 0.02), as shown in Fig. 2A; but CSS did not differ significantly between the non-PORT group and PORT group (HR = 0.92, 95% CI 0.85–1.01, *P* = 0.06), as shown in Fig. 2B. PSM was conducted to balance baseline characteristics between non-PORT and PORT groups (Supplementary Table S1). Following PSM, neither OS nor CSS did differ significantly between the non-PORT group and PORT group (OS HR = 1.08, 95%CI 0.98–1.19, *P* = 0.12; CSS HR = 1.10, 95%CI 0.99–1.23, *P* = 0.07) in unselected patients with surgically treated stage IIIA-N2 NSCLC, as shown in Fig. 2C, D. Furthermore, we found that PORT did not contribute to a survival benefit in patients with stage IIIA-N2 NSCLC diagnosed with adenocarcinoma, squamous, large cell, respectively, respectively (Supplementary Figure S1), and similar results were in patients with stage IIIA-N2 NSCLC underwent lobectomy, pneumonectomy and sublobectomy, respectively (Supplementary Figure S2).

## Discussion

Although a previous study reported that the use of PORT has declined for surgically treated stage IIIA-N2 NSCLC [11], we found that the number of surgically treated stage IIIA-N2 NSCLC following PORT was still large. However the role of PORT in patients with surgically treated stage IIIA-N2 NSCLC remains controversial [3, 4]. Consequently, there is a compelling need to explore the use of PORT on the survival of patients with stage IIIA-N2 NSCLC.

In previous studies, 20–60% patients with pathological N2 NSCLC had a locoregional recurrence [6, 12]. It is widely accepted that PORT contributes significantly to local–regional control in patients with surgically treated stage IIIA-N2 NSCLC [3, 4, 6, 7]. Therefore, PORT has been considered a strategy to improve outcomes by reducing the risk of local recurrence in patients with surgically treated stage III-N2 NSCLCT [6]. But PORT does not seem to improve OS in patients with surgically treated stage III-N2 NSCLCT [7, 13]. There are some possible reasons for the contradiction. Firstly, the benefit of PORT might be outweighed by itself as PORT naturally increases adverse events, such as cardiopulmonary toxicity [3, 8, 9]. However, several recent studies found that PORT might not contribute to an increase in the hazard for cardiac-related mortality [9, 14, 15]. A SEER population-based study reported no statistically significant difference in cardiac-related mortality between the PORT and non-PORT groups in stage IIIA-N2 NSCLC patients in all periods [9]. In the PORT-C trial, due to modern radiation techniques, such as IMRT, there were no radiotherapy-related grade 4 or 5 adverse events, and only 0.7% of patients had grade 3 radiation pneumonitis. With such low toxic effects, PORT still did not improve OS and DFS for patients with surgically treated stage IIIA-N2 NSCLC compared with the non-PORT group [5]. Secondly, because stage IIIA-N2 NSCLC is a systemic disease, it may be meaningless of local–regional control from PORT for patients with surgically treated stage IIIA-N2 NSCLC. The benefit of local–regional control is not equal to a survival benefit.

Patients with stage IIIA-N2 NSCLC in previous studies mostly were based on the former edition of TNM staging, but the edition of TNM staging have been re-defined. In this study patients were accurately diagnosed with primary IIIA-N2 NSCLC according to the latest 8th edition of TNM staging system, so the results in this study are more consistent with current clinical practice. Besides OS, CSS that only NSCLC-related death other than other causes was censored considered as endpoint was also taken into consideration to minimize the impact of other factors, such as cardiac-related mortality. Furthermore, PSM was performed to reduce the bias caused by selection, meanwhile both Cox multivariate analysis and PSM confirmed that PORT did not significantly improve OS or CSS in patients with IIIA-N2 NSCLC.

This study did not completely deny the application of PORT in surgically treated stage IIIA-N2 NSCLC. Although we do believe that PORT should have a survival benefit in selected patients with unique features, such as multiple N2 stations, a larger number of lymph nodes involvement, a bulky disease and high lymph node ratio (LNR) [16–22], the details or cut-off value of particular features have not yet come to a unified definition. Constructing a risk model, such as patient prognostic scores, might be a more potential candidate to select the proper patients with surgically treated stage IIIA-N2 NSCLC for PORT [12, 20]. Therefore, further studies exploring which patients with surgically treated stage IIIA-N2 NSCLC might optimally have a survival benefit from PORT are required.

There were several limitations in our study. First, potential selection bias cannot be excluded due to its retrospective nature. Second, due to the lack of the related details in SEER database, several important issues were still suspended, such as the PORT timing (concomitant, sequential, or alone) analysis, the N2 surgical resection (clinical N0, limited disease, single station disease, salvage surgery) and the metastatic station number: N2a1 (a single metastatic station with no hilar involvement), N2a2 (a single metastatic station with hilar involvement), and N2b (multiple metastatic stations) [23, 24]. Third, recent treatments, like molecular targeted therapy and cancer immunotherapy, play a vital role in treating patients with surgically treated stage IIIA-N2 NSCLC. Therefore, the impact of molecular targeted therapy and cancer immunotherapy cannot be excluded from this study due to the lack of data in the SEER database.

Generally, we found that PORT did not contribute to a survival benefit in unselected patients with surgically treated stage IIIA-N2 NSCLC. Further prospective randomized controlled trials are needed to confirm the findings.

### Supplementary Information

**Additional file 1:** **Supplementary Table S1.** Characteristics of patients with stage IIIA-N2 NSCLC after PSM.

**Additional file 2: Supplementary Figure S1.** PORT did not contribute to a survival benefit in patients with stage IIIA-N2 NSCLC diagnosed with adenocarcinoma, squamous, large cell, respectively, respectively.

**Additional file 3: Supplementary Figure S2.** Patients with stage IIIA-N2 NSCLC underwent lobectomy, pneumonectomy and sublobectomy, respectively.

## Data Availability

This study was subject to a Data Use Agreement with NCI. SEER data is publicly available and de-identified.
