# Correction: The high-risk features and effect of postoperative radiotherapy on survival for patients with surgically treated stage IIIA-N2 non-small cell lung cancer

**DOI:** 10.1186/s12957-023-03263-8

**Published:** 2024-01-03

**Authors:** Minxia Zhu, Shaomin Li, Liyue Yuan, Shiyuan Liu, Jianzhong Li, Danjie Zhang, Jia Chen, Jiantao Jiang, Zhengshui Xu

**Affiliations:** 1 Department of Thoracic Surgery, The Second Affiliated Hospital of Xi’an Jiaotong University, 157th Xiwu Road, Xincheng Distric, Xi’an, 710004 Shaanxi China; 2 Department of Thoracic Surgery, The First Affiliated Hospital of Wenzhou Medical University, Wenzhou, 325000 Zhejiang China; 3 Department of Endocrinology, The First Affiliated Hospital of Xi’an Jiaotong University, Xi’an, 710061 Shaanxi China; 4 Department of Cardiovascular Surgery, Xijing Hospital, Fourth Military Medical University, Xi’an, 710032 Shaanxi China

**Correction: World J Surg Oncol 21, 238 (2023)**

**https://doi.org/10.1186/s12957-023-03093-8**

Following publication of the original article [1], the authors reported an error in Fig. 1 and Supp. Figure S2.

The original article has been updated to correct this.
