# Supplementary material for: The high-risk features and effect of postoperative radiotherapy on survival for patients with surgically treated stage IIIA-N2 non-small cell lung cancer
Source: World J Surg Oncol. 2023 Aug 4;21:238. doi: 10.1186/s12957-023-03093-8 (PMC10401779; doi:10.1186/s12957-023-03093-8)
Supplement: Supplementary file 1 — Additional file 1: Supplementary Table S1. Characteristics of patients with stage IIIA-N2 NSCLC after PSM. [file 12957_2023_3093_MOESM1_ESM.docx]

| **Suppl. Table S1** Characteristics of patients with stage IIIA-N2 NSCLC after PSM | | | |
| --- | --- | --- | --- |
| Characteristic | non-PORT | PORT | *P*-value (χ2) |
| n | 1277 | 1277 |  |
| Age, n (%) |  |  | 0.91 |
| ＜65 | 569 (22.3%) | 565 (22.1%) | |
| ≥65 | 708 (27.7%) | 712 (27.9%) | |
| Sex, n (%) |  |  | 0.97 |
| Female | 708 (27.7%) | 710 (27.8%) | |
| Male | 569 (22.3%) | 567 (22.2%) | |
| Year of diagnosis, n (%) | | | 0.58 |
| 2000-2009 | 596 (23.3%) | 581 (22.7%) | |
| 2010-2019 | 681 (26.7%) | 696 (27.3%) | |
| Race, n (%) | |  | 0.75 |
| Other | 213 (8.3%) | 220 (8.6%) |  |
| White | 1064 (41.7%) | 1057 (41.4%) | |
| Primary site, n (%) | |  | 0.38 |
| Lower lobe | 374 (14.6%) | 344 (13.5%) | |
| Middle lobe | 48 (1.9%) | 54 (2.1%) |  |
| Upper lobe | 855 (33.5%) | 879 (34.4%) | |
| Laterality, n (%) | |  | 0.87 |
| Left | 582 (22.8%) | 587 (23%) |  |
| Right | 695 (27.2%) | 690 (27%) |  |
| Histologic Type, n (%) | | | 0.81 |
| Large cell | 25 (1%) | 27 (1.1%) |  |
| LUAD | 981 (38.4%) | 976 (38.2%) | |
| LUSC | 188 (7.4%) | 200 (7.8%) |  |
| Other | 83 (3.2%) | 74 (2.9%) |  |
| T stage, n (%) | |  | 0.90 |
| T1 | 502 (19.7%) | 506 (19.8%) | |
| T2 | 775 (30.3%) | 771 (30.2%) | |
| Surgery, n (%) | |  | 0.59 |
| Lobectomy | 1131 (44.3%) | 1125 (44%) | |
| Pneumonectomy | 47 (1.8%) | 57 (2.2%) |  |
| Sublobectomy | 99 (3.9%) | 95 (3.7%) |  |
| Chemotherapy, n (%) | | | 1.00 |
| No | 233 (9.1%) | 233 (9.1%) |  |
| Yes | 1044 (40.9%) | 1044 (40.9%) | |
| Regional nodes positive, n (%) | | | 0.77 |
| ＜4 | 839 (32.9%) | 831 (32.5%) | |
| ≥4 | 438 (17.1%) | 446 (17.5%) | |
