# Supplementary figures and images for: The high-risk features and effect of postoperative radiotherapy on survival for patients with surgically treated stage IIIA-N2 non-small cell lung cancer
Source: World J Surg Oncol. 2023 Aug 4;21:238. doi: 10.1186/s12957-023-03093-8 (PMC10401779; doi:10.1186/s12957-023-03093-8)

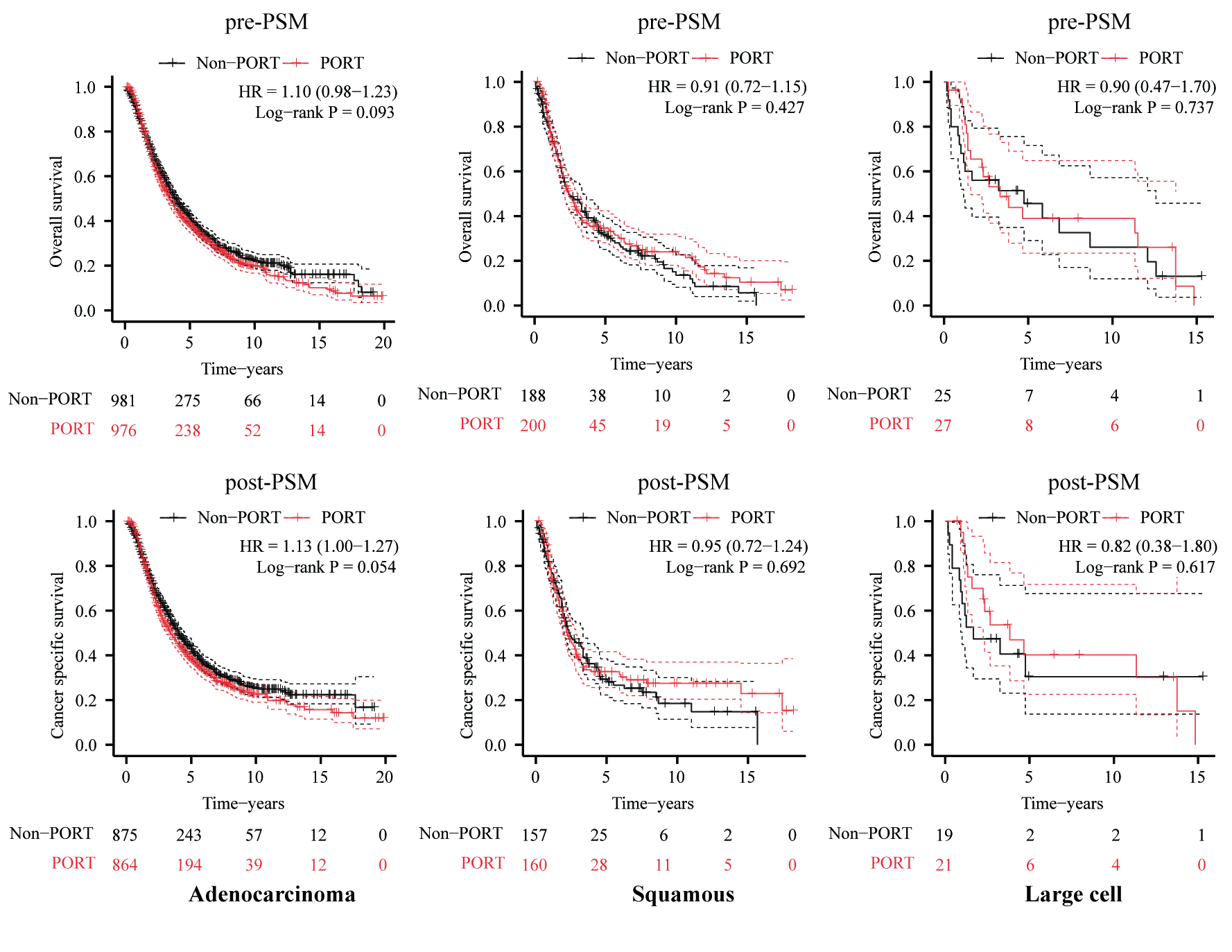

Supplement: Supplementary file 2 — Additional file 2: Supplementary Figure S1. PORT did not contribute to a survival benefit in patients with stage IIIA-N2 NSCLC diagnosed with adenocarcinoma, squamous, large cell, respectively, respectively. [file 12957_2023_3093_MOESM2_ESM.tif]

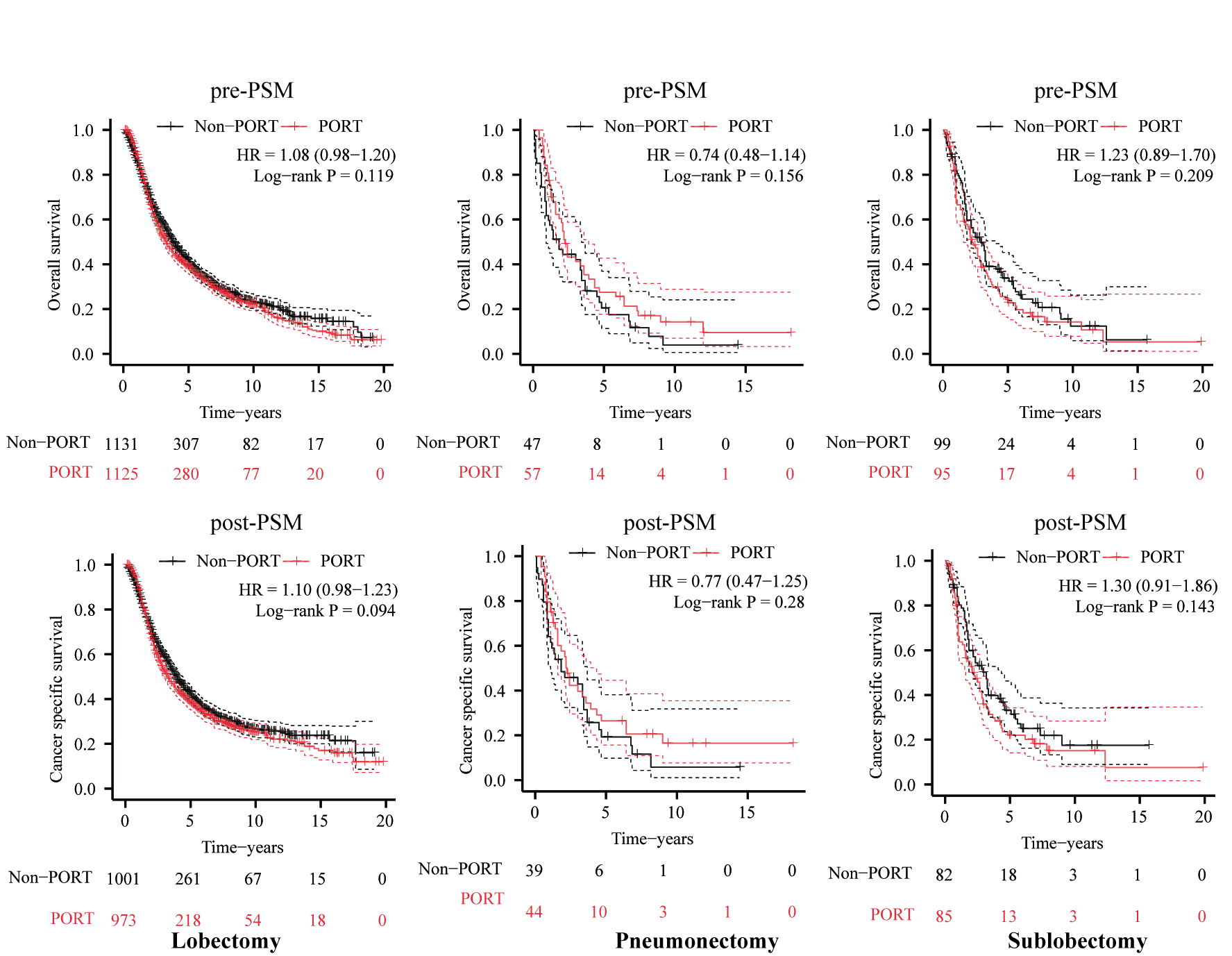

Supplement: Supplementary file 3 — Additional file 3: Supplementary Figure S2. Patients with stage IIIA-N2 NSCLC underwent lobectomy, pneumonectomy and sublobectomy, respectively. [file 12957_2023_3093_MOESM3_ESM.tif]
